# FDG positron emission tomography imaging and ctDNA detection as an early dynamic biomarker of everolimus efficacy in advanced luminal breast cancer

**DOI:** 10.1038/s41523-021-00331-8

**Published:** 2021-09-21

**Authors:** Andrea Gombos, David Venet, Lieveke Ameye, Peter Vuylsteke, Patrick Neven, Vincent Richard, Francois P. Duhoux, Jean-Francois Laes, Françoise Rothe, Christos Sotiriou, Marianne Paesmans, Ahmad Awada, Thomas Guiot, Patrick Flamen, Martine Piccart-Gebhart, Michail Ignatiadis, Géraldine Gebhart

**Affiliations:** 1 Medical Oncology Department, Institut Jules Bordet and Université Libre de Bruxelles (ULB), Brussels, Belgium; 2 Université Libre de Bruxelles, Brussels, Belgium; 3 Breast Cancer Translational Research Laboratory, Institut Jules Bordet and Université Libre de Bruxelles (ULB), Brussels, Belgium; 4 Statistical Department, Institut Jules Bordet, Université Libre de Bruxelles (ULB), Brussels, Belgium; 5 Medical Oncology, UCLouvain, CHU Namur Site Sainte-Elisabeth, Namur, Belgium; 6 University of Botswana, Private Bag UB 0022, Gabarone, Botswana; 7 Multidisciplinary Breast Center and Department of Gynecology and Obstetrics, UZ Leuven, KU Leuven, Leuven, Belgium; 8 Medical Oncology CHU Ambroise Paré, Mons, Belgium; 9 Medical Oncology Cliniques Universitaires Saint-Luc, Brussels, Belgium; 10 OncoDNA, Gosselies, Belgium; 11 Nuclear Medicine, Institut Jules Bordet, Brussels, Belgium

**Keywords:** Breast cancer, Breast cancer

## Abstract

Biomarkers to identify patients without benefit from adding everolimus to endocrine treatment in metastatic breast cancer (MBC) are needed. We report the results of the Pearl trial conducted in five Belgian centers assessing ^18^F-FDG-PET/CT non-response (*n* = 45) and ctDNA detection (*n* = 46) after 14 days of exemestane-everolimus (EXE-EVE) to identify MBC patients who will not benefit. The metabolic non-response rate was 66.6%. Median PFS in non-responding patients (using as cut-off 25% for SUVmax decrease) was 3.1 months compared to 6.0 months in those showing response (HR: 0.77, 95% CI: 0.40–1.50, *p* = 0.44). The difference was significant when using a “post-hoc” cut-off of 15% (PFS 2.2 months vs 6.4 months). ctDNA detection at D14 was associated with PFS: 2.1 months vs 5.0 months (HR-2.5, 95% CI: 1.3–5.0, *p* = 0.012). Detection of ctDNA and/or the absence of ^18^F-FDG-PET/CT response after 14 days of EXE-EVE identifies patients with a low probability of benefiting from treatment. Independent validation is needed.

## Introduction

Everolimus is a rapamycin derivate that inhibits m-TOR by binding to mTORC1. The combination of everolimus and an endocrine agent has been studied in several randomized trials in patients with locally advanced and metastatic breast cancer, all showing consistent improvement in progression-free survival (PFS)^1–3^.

The phase III BOLERO-2 registration trial (*n* = 724) was conducted in postmenopausal patients diagnosed with endocrine receptor-positive (ER + ), HER2-negative (HER2-) metastatic breast cancer (MBC) whose disease progressed during or after treatment with non-steroidal aromatase inhibitors and showed a significantly improved PFS with exemestane + everolimus (EXE + EVE) (10.6 months) versus exemestane alone (4.1 months), HR: 0.43; 95% CI: 0.35–0.54; *P* < 0.001. This benefit comes at a price of increased toxicity, altering quality of life (stomatitis: 56%; skin rash: 36%; fatigue: 33%; diarrhea: 30%; nausea: 27%; interstitial pneumonitis: 12%)^2^.

Translational research efforts conducted so far on primary/metastatic tumor tissue or baseline circulating tumor DNA (ctDNA) samples have failed to identify clinically useful biomarkers of benefit, except for p4EBP1. This, however, requires metastatic biopsies before starting treatment^4–7^. None of these translational studies have assessed the association between early changes in these biomarkers and the benefit of adding everolimus to exemestane.

It is evident that the EXE + EVE combination plays a role in the treatment of patients with endocrine-resistant metastatic breast cancer. However, it is unclear in which patients the clinical benefit justifies the potential toxicity. Considering the high rate of adverse events and high cost associated with this treatment, the early identification of patients who will not derive any benefit is of utmost importance, both medically and financially.

We therefore developed the phase II PEARL study (PET imaging as a biomarker of Everolimus Added value in hormone Refractory postmenopausaL women, NCT 02028364) to evaluate the clinical utility of an early positron emission tomography with 2-deoxy-2-[fluorine-18]fluoro- D-glucose integrated with computed tomography imaging (^18^F-FDG-PET/CT) assessment (performed on day 14 [D14] after treatment initiation) as a biomarker to select patients with ER + , HER2− metastatic breast cancer who will not benefit from this treatment combination. At the same time, we studied ctDNA to identify and further characterize non-responding patients using serial plasma samples. We hypothesized that a combination of biomarkers would be associated with an improved negative predictive value in terms of predicting the lack of benefit from EXE + EVE.

## Results

### Patients

Sixty-four patients were screened to include 47 evaluable patients according to the ^18^F-FDG-PET/CT eligibility criteria in 5 centers across Belgium between February 2014 and June 2018. Forty-five patients were included in the final ^18^F-FDG-PET/CT analysis. Two patients were excluded from the metabolic response analysis because they stopped everolimus at 3 and 8 days respectively before the D14 ^18^F-FDG-PET/CT was performed. (Supplementary Fig. 1a). The median follow-up was 17.4 months. Since data on ^18^F-FDG-PET/CT in patients treated with everolimus were scarce, we first conducted a pilot phase (*n* = 27, early ^18^F-FDG-PET/CT realized 14 and 28 days respectively after treatment initiation). Recruitment was held between June 2016 and June 2017 after the first 27 patients in order to perform an interim analysis of the pilot phase and to choose the most suitable time point for the early ^18^F-FDG-PET/CT assessment. In the pilot phase, metabolic non-responders represented 59% of the population at both time points (Kappa coefficient for metabolic response concordance: 0.85; mean standardized uptake value [SUV] decrease at D14: 26 %). Given the high concordance between the D14 and D28 results, we chose the D14 assessment for the second phase.

Concerning the ctDNA analysis, 46 out of 47 patients were included, since baseline plasma samples were missing for one patient (Supplementary Fig. 1b). A total of 121 sequential plasma samples were analyzed. The ctDNA analysis population comprised the following: 46 patients at baseline, 42 patients on D14, and 33 patients at progression (Supplementary Fig. 1b). Differences in the number of plasma samples analyzed at different time points are related to missing samples.

The patients were all postmenopausal, with the mean age at inclusion being 57 years (± 11 years). Histological characteristics of the primary tumor were locally assessed. The majority of patients (83%) had invasive ductal carcinoma of non-special type, and 62% of the tumors were luminal B using immunohistochemistry (IHC) for Ki 67 (Ki 67≥15%)^8^. Seventy-four percent of the patients had visceral disease (lung or liver), whereas 13% had bone-only metastases. Cyclin-dependent kinase 4/6 (CDK 4/6) inhibitors were not yet reimbursed during most of the recruitment period; thus, only 12 patients (26%) benefited from this treatment before inclusion. More than half of the patients had received at least one previous line of chemotherapy (30% one line and 28% ≥2 lines), and almost all of them had received prior endocrine treatments (68% ≥2 lines). Seventy percent had the disease previously sensitive to non-steroidal aromatase inhibitors. The detailed patient characteristics (*n* = 47) are listed in Table 1.

**Table 1:** Detailed patient characteristics (*n* = 47).

*Age*		
Mean/IQR (years)	57.1 ± 13.4	
*ECOG PS*		
0	23	49%
1	23	49%
2	1	2%
*Histology*		
Invasive ductal	38	83%
Invasive lobular	8	17%
Unknown	1	
*Grade*		
G1	8	22%
G2	17	47%
G3	11	31%
Unknown	11	
*Current disease status*		
Metastatic	45	96%
Lung or liver	35	74%
Bone only	6	13%
Other	4	9%
Locally advanced	2	4%
*N Metastatic sites*		
0	2	4%
1–2	24	51%
≥3	21	45%
*KI-67 (primary)*		
<10%	5	14%
10–15%	9	24%
16–25%	6	16%
>25%	17	46%
Unknown	10	
*Prior CDK 4/6*		
No	35	74%
Yes	12	26%
*Number of lines CT in advanced setting*		
0	20	43%
1	14	30%
≥2	13	28%
*Number of lines ET in advanced setting*		
0	2	4%
1	13	28%
≥2	32	68%
*NSAI sensitive*		
No	13	30%
Yes	34	70%

Baseline patient demographics (*n* = 47).

Represents baseline characteristics of the 47 patients considered to be eligible according to the 18F-FDG-PET/CT eligibility criteria (details in Method section).

IOR = interquartile range; ECOG PS = Eastern Cooperative Oncology Group performance status, ranging from 0 to 5, 0 indicating that the patient is fully active and able to carry on all pre-disease performance without restriction; at 1, restricted in physically strenuous activity but ambulatory and able to carry out work of a light or sedentary nature; at 2, ambulatory and capable of all self-care but unable to carry out any work activities; up to about more than 50% of waking hours; N Metastatic sites = number of metastatic sites; Ki-67 = cell proliferation marker (immunohistochemistry); CDK 4/6 = cyclin-dependent kinase 4/6 inhibitor; CT = chemotherapy; ET = endocrine therapy; NSAI = non-steroidal aromatase inhibitor; sensitive is defined as relapse ≥2 years after the end of an NSAI in the adjuvant setting, or ≥6 months of treatment in the metastatic setting.

### ^18^F-FDG-PET/CT response on D14 and patient outcome

**Fig. 1:**
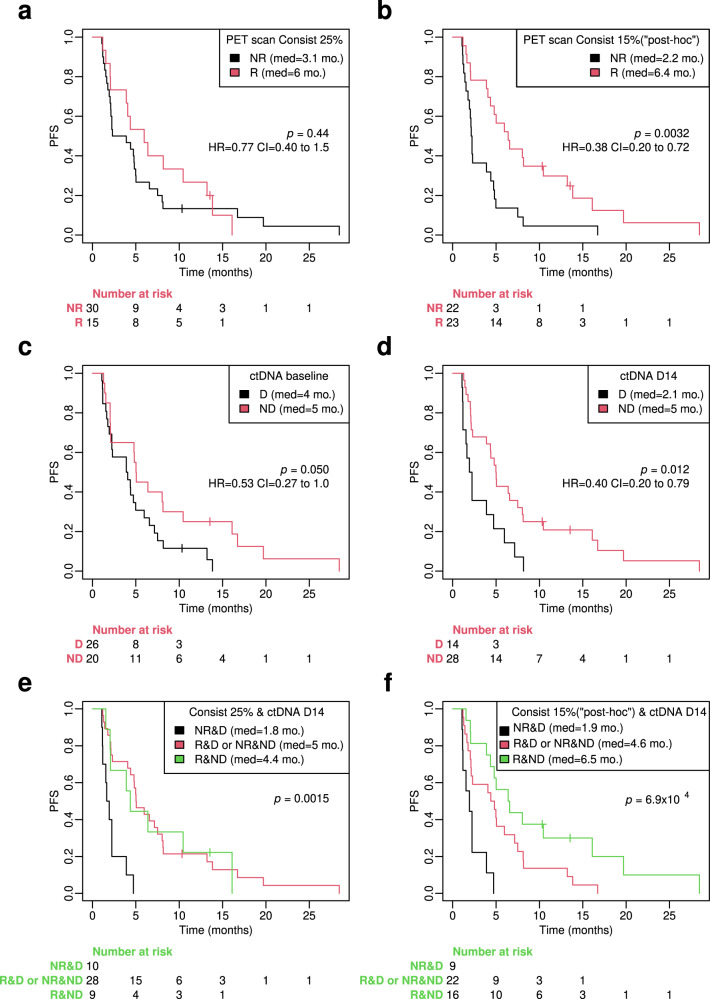
Kaplan–Meier plots of PFS according to ^18^F-FDG-PET/CT response and ctDNA detection 14 days after the start of treatment with exemestane-everolimus. **a** PFS plots according to ^18^F-FDG-PET/CT response on D14: patients with >25% homogenous decrease in maximum standardized uptake value (SUVmax) in all target lesions are considered as “responders”. **b** PFS plots according to ^18^F-FDG-PET/CT response on D14: patients with *a* > 15 % homogenous decrease in maximum standardized uptake value (SUVmax) in all target lesions are considered as “responders”. This is a “post-hoc” analysis, initially not scheduled by the study protocol. **c** PFS according to ctDNA detection at baseline (40-gene targeted gene sequencing panel was realized in all plasma samples). **d** PFS according to ctDNA detection after 14 days of treatment with exemestane-everolimus. **e** PFS according to the combined analysis of ctDNA detection and ^18^F-FDG-PET/CT response when patients with >25% homogenous decrease in maximum standardized uptake value (SUVmax) in all target lesions are considered as “responders”. **f** Combined analysis of ctDNA detection and ^18^F-FDG-PET/CT response when patients with >15% homogenous decrease in maximum standardized uptake value (SUVmax) in all target lesions are considered as “responders”. The 15% cut-off is a “post-hoc” analysis, initially not scheduled by the study protocol. Log-Rank P values and LogRank Hazard ratios and 95% Confidence intervals for LogRank Hazard ratios for each test are displayed in each corresponding panel. For each group, the number at risk is presented under the *X*-axis. Consist criteria of ^18^F-FDG-PET/CT response is described Methods section, R = ^18^F-FDG-PET/CT responder; NR = ^18^F-FDG-PET/CT non-responder; ctDNA = circulating tumor DNA; D = ctDNA detected; ND = ctDNA not detected; D14 = 14 days after starting treatment with exemestane-everolimus.

The metabolic non-response rate according to the criteria initially defined in the protocol (i.e., patients with a SUVmax reduction of more than 25% in all lesions classified as responders) was 66.6%: 30 patients out of the 45 included in the final PFS analysis were non-responders. There was no statistically significant difference in outcome between those patients who were considered responders and those who were not. The median PFS for ^18^F-FDG-PET/CT response was 6.0 months compared to 3.1 months for non-response, but this difference was non-significant with an HR of 0.77 (95% CI, 0.40–1.50), *p* = 0.44 (Fig. 1a). Results were similar in the intention to treat (ITT) population (*n* = 47), including those two patients who stopped everolimus before D14 ^18^F-FDG-PET/CT (data not shown).

However, the 25% threshold to define response has previously mainly been used in patients receiving chemotherapy and to assess late response^9^. We therefore retrospectively looked for a metabolic response cut-off that could be more adapted to a targeted treatment such as everolimus (Supplementary Fig. 3a, c). By using 15% as a cut-off, probably a more accurate one for an early response evaluation already after 14 days of targeted therapy, the median PFS of responders (*n* = 23) was 6.4 months versus 2.2 months of non-responders, *p* = 0.0032, HR-0.38 (95% CI 0.20–0.72) (Fig. 1b).

The results are similar if we depict the PFS according to different classes of Consist response. Consist responders class 1 (i.e. no evidence of metabolic non-responsive lesion) have a significantly better prognosis than non-responders (classes 2–4) when we use the 15% SUVmax decrease as a cut-off to define response (Supplementary Fig. 2). As initially defined in the protocol, ^18^F-FDG-PET/CT response was also assessed by PERCIST criteria. The prognostic value of early PET response according to this criterion is shown in Supplementary Fig. 3b, d.

### Combined analysis of ^18^F-FDG-PET/CT response and ctDNA detection on D14 and patient outcome

As a next step, we assessed whether the combination of ctDNA detection and absence of metabolic response with ^18^F-FDG-PET/CT on D14 can better identify those patients who will not derive benefit from the EXE-EVE combination than either method alone. ctDNA was detected in 26 out of 46 patients at baseline (56.5%) and in 14 out of 42 patients on D14 (33.3%).

The median PFS on EXE-EVE was only 1.8 months in patients with no metabolic response and detectable ctDNA on D14 (Fig. 1e). By contrast, patients with metabolic response and no detectable ctDNA at D14 had a median PFS of 4.4 months, *p* = 0.0015 (Fig. 1e). The difference between these two groups of patients is even more obvious when we use 15% reduction of SUVmax to define ^18^F-FDG-PET response (median PFS 6.5 months versus 1.9 months, *p* = 6.9 × 10^−4^) (Fig. 1f).

**Fig. 2:**
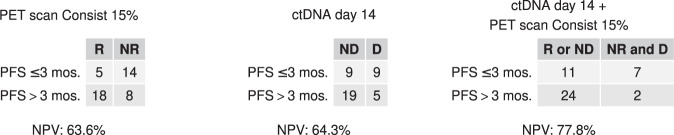
Negative predictive value (NPV) of 18F-FDG-PET/CT metabolic non-response and ctDNA detection on D14 to predict early disease progression, after only 3 months of treatment with exemestane-everolimus. NPV (expressed in %) is displayed below each panel. PFS progression-free survival, NPV negative predictive value; R = ^18^F-FDG-PET/CT responder; NR = ^18^F-FDG-PET/CT non-responder; ctDNA = circulating tumor DNA; D-ctDNA detected; ND-ctDNA not detected; D14 = 14 days after starting treatment with exemestane-everolmius.

We also assessed the value of ^18^F-FDG-PET non-response (using Consist 15%), ctDNA positivity on D14, and the combination of both to identify patients with rapid disease progression (PFS of less than 3 months) using the negative predictive value. The negative predictive value of PET scan non-response, ctDNA detection on D14, and the combination of both was 63.6% (14/22 patients), 64.3% (9/14 patients), and 77.8% (7/9 patients), respectively (Fig. 2).

### Early ctDNA dynamics and correlation with patient outcome

As previously mentioned with the 40-gene panel we used (Supplementary Table 1) ctDNA was detected in 26 out of 46 patients at baseline (56.5%) and in 14 out of 42 patients on D14 (33.3%). Next, we analyzed whether ctDNA detection alone (yes vs. no) at baseline and at D14 has an impact on patient outcome when treated with EXE-EVE. Median PFS in patients with versus without detected ctDNA at baseline was 5 versus 4 months, *p* = 0.050, HR-1.9 (95% CI: 0.99–3.6) (Fig. 1c). More importantly, median PFS in patients with ctDNA detection at D14 was significantly lower than in patients without ctDNA detection (2.1 versus 5 months, respectively, HR 2.5 [95% CI: 1.3–5.0 *p* = 0.012] (Fig. 1d).

When performing the same analysis at D14, but using only data of patients in whom ctDNA was detected at baseline and had available sample on D14 (*n* = 23), similarly PFS doubles when ctDNA in not detectable on D14 (4.4 months vs. 2.1 months, HR 0.51, 95% CI: 1.3–5.0, *p* = 0.12). (Supplementary Fig. 7) However the difference is not significant and this data should be interpreted with caution given the small number of patients in both groups.

**Fig. 3:**
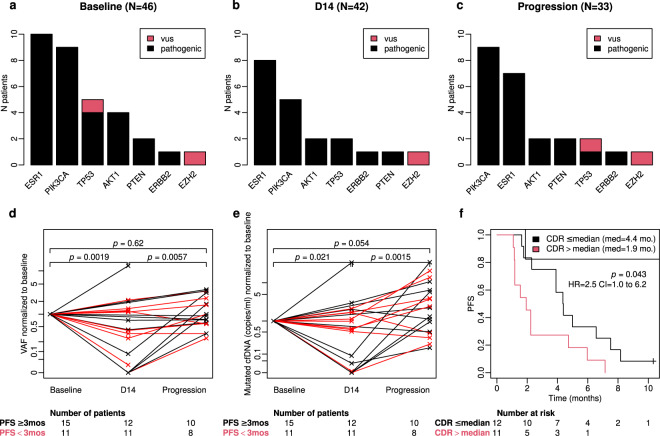
Dynamic assessment of mutations in ctDNA during treatment with exemestane-everolimus. **a**, **b**, **c** Somatic mutations detected in ctDNA at three different time-points during treatment with exemestane-everolimus: baseline (**a**), 14 days after treatment initiation (**b**), and at progression (**c**). vus = variant of unknown significance; prog = progression. d-Variant allele frequency (VAF) of SNVs (single nucleotide variants) and their evolution between three different time points (baseline, D14, and progression). VAF values are normalized to baseline. Black lines depict patients who had a PFS ≥ 3 months; red lines depict patients who have a PFS <3 months. The number of patients in each group and time-point are presented under the *X*-axis. **e**: Evolution of mutant cfDNA (cell-free DNA) copies (expressed in copies/ml of plasma) during exemestane-everolmus treatment. We note a significant decrease of mutated cfDNA between baseline and D14. Black lines depict patients who had a PFS ≥3 months; red lines depict patients who have a PFS < 3 months. The number of patients in each group and time-point are presented under the *X*-axis. **f**: Kaplan–Meier plots of PFS according to Circulating cfDNA ratio (CDR). CDR is a ratio of cfDNA copies/ml of all somatic mutations detected on D14 and baseline. Log-Rank P values and LogRank Hazard ratios and 95% Confidence intervals for LogRank Hazard ratios for each test are displayed in each corresponding panel. For each group the number at risk is presented under the *X*-axis.

Furthermore, we assessed whether the circulating DNA ratio (CDR), defined as a ratio of mutated copies/ml between D14 and baseline as previously described by O’Leary et al.^10^, is associated with PFS in the group of patients with detectable ctDNA at baseline and available plasma samples at both time points. We observed a statistically significant better PFS for patients with CDR levels below or equal to the median value (≤0.061): 4.4 month vs. 1.9 month, HR 2.5, 95% CI: 1.0–6.2, *p* = 0.043 (Fig. 3f).

Next, we evaluated whether ctDNA mutation variant allele frequency (VAF) (0 versus 10–20% vs. ≥20%) at baseline and at D14 was associated with PFS. We found that increasing ctDNA mutation VAF was associated with worse PFS at both time points (Supplementary Fig. 5).

### ctDNA mutational landscape and evolution during treatment

The most frequently detected hotspot somatic single nucleotide variants, SNVs (to be referred as somatic mutations from now on in the text), in ctDNA occurred in the *ESR1, PIK3CA, TP53*, and *AKT1* genes. Mutations in the *PTEN* and *ERBB2* genes were less frequently detected (Fig. 3a). Most patients had 1 detectable mutation in plasma cell-free DNA. Only 5 patients at baseline and 4 patients on D14 had 2 detectable mutations (each time in different genes). None of the patients presented more than 2 mutations. When we focused on plasma samples with detectable ctDNA, we observed no major differences in the mutational landscape between the different time-points when sequencing was performed: baseline, D14, and progression (Fig. 3a, c).

Detected somatic mutations, the ctDNA mutation VAF, and the number of total and mutated copies/ml of plasma analyzed at three different time points (baseline, D14, and progression) for each individual patient are listed in Supplementary Table 2.

Of note, there was a statistically significant decrease in ctDNA between baseline and D14 using either mutated VAF or mutated copies/ml for quantification We also observed an increase in ctDNA between D14 and progression (Fig. 3d, e).

Finally, we looked at the prognostic value of ctDNA mutations in the two most frequently mutated genes in our cohort: *ESR1* and *PIK3CA*. Only *ESR1* mutations, but not *PIK3CA*, had a significant impact on ^18^F-FDG-PET/CT response. Interestingly, none of the patients in whom an *ESR1* mutation was detected at baseline (*n* = 10) had a metabolic response at D14 when using the initially defined 25% cut-off (Supplementary Fig. 4a–d). There was no association between the detection of either *ESR1* or *PIK3CA* mutations in baseline plasma samples and PFS (Supplementary Fig. 4e, f). *ESR1* mutations in ctDNA were detected in 10 out of 46 patients at baseline; 6 patients had the D538G mutation, 2 the Y537N mutation, and 2 the Y537S mutation. The number of different types of *ESR1* mutations is too low to determine any association between specific mutations and clinical outcomes.

### Integration of ctDNA and ^18^F-FDG-PET/CT analysis in the landscape of other clinical and biological variables

**Fig. 4:**
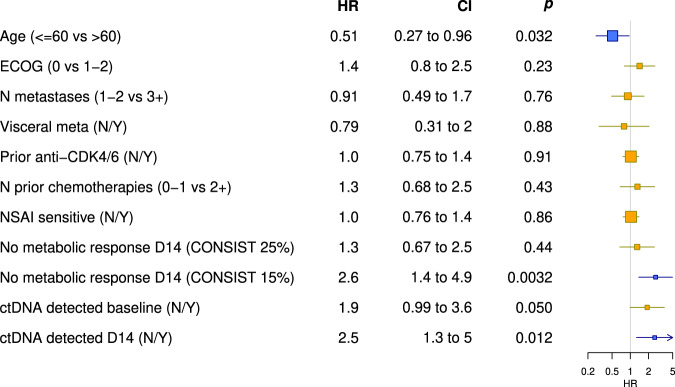
Impact of clinical and pathological characteristics, ^18^F-FDG-PET/CT response and ctDNA detection on PFS (univariate analysis). Blue boxes represent parameters that have a significant impact on PFS. Yellow boxes represent parameters which doesn’t have significant impact on PFS. Horizontal lines represent CI. *X*-axis represents HR. CI: 95% confidence interval, p: *p* value, HR: hazard ratio. ECOG = Eastern Cooperative Oncology Group performance status, ranging from 0 to 5, and indicating that the patient is at 0, fully active able to carry on all pre-disease performance without restriction; at 1,restricted in physically strenuous activity but ambulatory and able to carry out work of a light or sedentary nature; at 2, 2 - ambulatory and capable of all self-care but unable to carry out any work activities; up about more than 50% of waking hours; N metastasis = number of metastatic organ sites involved, Visceral meta = presence of visceral metastasis, N = no, Y = yes, N prior chemotherapies = number of prior chemotherapy regimens used for the treatment of advanced breast cancer, NSAI = non-steroidal aromatase inhibitor; sensitive is defined as relapse ≥2 years after the end of a NSAI in adjuvant setting or ≥ 6 months treatment in metastatic setting, Consist 25% = <25% homogenous decrease in maximum standardized uptake value (SUVmax) in all target lesions are considered as “non-responders”, Consist 15% = <15% homogenous decrease in maximum standardized uptake value (SUVmax) in all target lesions are considered as “non-responders”.

When we performed a univariate analysis of the above-described parameters and other clinical and biological characteristics, young age (≤60 years), ctDNA detection at D14, and the absence of metabolic response using the threshold of 15% had a significantly negative impact on outcome (Fig. 4). We did not collect data on breast cancer-specific tumor markers (CA 15-3), and thus this variable could not be integrated into the analysis. Other clinical characteristics and previous treatments received were not associated with a worse PFS in this population.

As a final step, we assessed if the number of metastatic lesions identified on baseline ^18^F-FDG-PET/CT or the metabolically active tumor volume (MATV) as a surrogate of disease burden define the patient’s outcome. For both parameters, we evaluated if a specific cut-off can delineate patients with a worse prognosis (Supplementary Fig. 8b, d). Patients with an MATV >100 cm^3^ had a significantly worse outcome than those with MATV ≤100 cm^3^ (Supplementary Fig. 8a). Similarly, we observed a PFS of only 1.9 months when more than 7 metastatic lesions were identified on baseline ^18^F-FDG-PET/CT compared to 5 months if the number of lesions was ≤7 (Supplementary Fig. 8c). ^18^F-FDG-PET/CT response was not influenced by MATV, while metabolic response tends to be worse if the number of lesions in high. (Supplementary fig. 8e–h).

## Discussion

In the PEARL study, we were able to demonstrate that patients with ER + , HER2- metastatic breast cancer whose early ^18^F-FDG-PET/CT shows lack a metabolic response and/or who have detectable ctDNA after 14 days of treatment with the EXE-EVE combination have substantially lower PFS than responding patients.

The overall clinical outcome in our trial is poorer than in the previously reported larger BOLERO-2 study, probably because our cohort had received more prior lines of treatment (28% of patients had had two or more lines of chemotherapy for metastatic breast cancer in PEARL, whereas only 26% of patients had received a single line of chemotherapy in the same setting in BOLERO-2, and multiple lines were not permitted)^2^.

Our primary hypothesis was that patients who do not show an early-^18^F-FDG-PET/CT response will have a significantly shorter PFS than those who are considered “early responders”. This way we aimed to identify already after two weeks of EXE-EVE treatment a clinical subgroup of patients whose disease will most probably progress by the time of their first radiological response assessment (at 3 months). Thus, these patients could interrupt treatment after only two weeks, avoiding their exposure to unnecessary toxicities and minimizing high costs for society.

We acknowledge that the initially established threshold to define ^18^F-FDG-PET/CT response was ambitious and inspired by previous data from patients treated with chemotherapy. We chose to use the so called Consist patient-based response method described for metastatic colorectal cancer^9^. This method defines as responders only those patients who have a homogenous decrease of SUVmax in all metastatic lesions^9^. Other methods such as PERCIST and EORTC consider only a limited number of operator-selected target lesions and might dismiss tumor heterogeneity and the emergence of resistant clones under treatment^11^. Using a 25% cut-off we were able to document an absolute difference of 3 months in PFS between PET responders and non-responders, which was not statistically significant, however.

Another trial, assessing early-^18^F-FDG-PET/CT response in breast cancer patients treated with double HER2-blockade (trastuzumab and lapatinib) in the neoadjuvant setting, selected as a threshold a 15% reduction of the SUVmax in all target lesions to define response^12^. This trial used early PET during a chemotherapy-free window. Similarly, in a trial looking at imaging biomarkers in a HER2-positive metastatic population treated with T-DM1, an early ^18^FDG-PET-CT was performed after one cycle of treatment and was shown to be highly correlated with the absence of RECIST 1.1 response after 3 cycles of treatment when using the 15% cut-off (negative predictive value of 83%)^13^.

According to these data, we retrospectively assessed in our cohort the threshold of 15% to define a metabolic response. This was probably more adapted to our patient population, which had been treated with a combination that would basically lead to a low probability of tumor shrinkage (9% in BOLERO-2)^2^. With this second method in a post-hoc analysis we were consequently able to demonstrate a statistically significant difference: the median PFS of responders was 6.4 months compared to only 2.2 months for non-responders. This cut-off had never been used previously in the ER + metastatic breast cancer population; our trial (PEARL) is the first to report these data in this type of breast cancer.

In future clinical trials, we would recommend a low cut-off (15%) to identify patients not benefiting from therapy as soon as two weeks after treatment initiation. If the trial aims to identify responding patients, the cut-off should be higher and might be more influenced by the type of breast cancer or therapy.

Secondarily, we assessed whether ctDNA detection at baseline or D14 or ctDNA changes between baseline and D14, were associated with PFS and whether this method could increase the negative predictive value of ^18^FDG-PET-CT to detect non-responders early on. With this aim, we performed a targeted gene sequencing of serial plasma samples. We were able to show that PFS in patients who still have detectable ctDNA after 14 days of treatment is particularly low: 2.1 months versus 5.0 months in those without detectable ctDNA (*p* = 0.012). The PFS of patients who showed no metabolic response (with a 15% cut-off) and the PFS of those who had ctDNA detection on D14 was similar. This suggests that using ctDNA detection after two weeks of treatment might also help to identify patients who are unlikely to benefit from EXE-EVE. To our knowledge, our study is the first to report sequential ctDNA monitoring in patients with metastatic breast cancer treated with EXE-EVE.

Early *PIK3CA* ctDNA dynamics as assessed by circulating DNA ratio (the ratio of mutated *PIK3CA* copies/ml on D15 relative to baseline) during treatment with fulvestrant and palbociclib (a CDK 4/6 inhibitor) was associated with PFS in a retrospective analysis of plasma samples collected in the PALOMA3 trial^10^. We defined CDR as a ratio of cfDNA copies/ml of all somatic mutations detected on D14 and baseline. In our study, as well, we observe a significantly better PFS for patients with CDR levels below the median value (0.061). These data should be interpreted with caution, however, considering the limited sample size in different groups. More recently, the PADA-1 phase III trial (*n* = 1017) showed that clearance of the *ESR1* mutations in ctDNA after 1 month of treatment with an aromatase inhibitor and palbociclib (a CDK 4/6 inhibitor) in patients with ER + , HER2- metastatic breast cancer was associated with improved prognosis in those patients who had detectable *ESR1* mutations when starting treatment. Median PFS was 24.1 months, compared to 7.4 months for patients who did not clear this particular mutation^14^. All these data suggest that longitudinal evaluation of ctDNA during treatment is probably more powerful and more accurate in predicting response and long-term outcome than baseline assessment alone.

From a clinical practice point of view, it would have been desirable to show a negative predictive value of at least 85% for one of the two dynamic tests or their combination, since most oncologists are likely to continue their patients’ therapy beyond D14 for a 20–25% chance of later response. According to our results, the negative predictive value for a PFS of less than 3 months when we use both tests together is 77.8%, meaning that there were still 2 out of 9 patients with no ^18^F-FDG-PET/CT response and with ctDNA detection on D14 who had a PFS of more than 3 months.

With the 40-gene targeted gene sequencing approach we used, we were able to detect at least one somatic mutation before starting the EXE-EVE combination in 26 out of 46 patients (56.5%). We acknowledge, that our panel is not breasted cancer-specific and could have missed some of the SNVs previously described in MBC tumor samples using whole exome or whole genome sequencing^15,16^. Thus ctDNA negativity may be attributable to the absence of ctDNA or to the absence of somatic mutation within the short panel of genes that were assessed. However, our panel includes the most frequently described clonal mutations in metastatic breast cancer such as *PIK3CA* and *TP53* as well as other genes that have been linked to resistance to endocrine and targeted therapy in ER + /HER2− breast cancer such as *ESR1, ERBB2, FGFR1, PTEN, AKT1*^17,18^.

As expected, the most frequently detected alterations were present in the *ESR1* and *PIK3CA* genes, found respectively in 21.7% and 19.6% of baseline samples. When we individually assess the prognostic value of these two most frequently mutated genes, PFS is not worse in patients with *ESR1* or *PIK3CA* mutations detected at baseline. However, given the small number of patients in each group, this analysis should also be interpreted with caution. It should be noted that the rate of *PIK3CA* mutations in our cohort is lower than in the BOLERO-2 population (43.3% in BOLERO-2 compared to 19.6% in our cohort). The droplet digital polymerase chain reaction (ddPCR) used in BOLERO-2 to identify hotspot mutations in three epitopes, *PIK3CA* H1047R, E545K, and E542K^5^, is probably more sensitive than the targeted gene sequencing approach applied to our samples. Detection of *PIK3CA* mutations in baseline cfDNA was not associated with a different benefit from the addition of everolimus to exemestane in the BOLERO-2 trial. When *ESR1* mutations were analyzed in the BOLERO-2 trial, it appeared that patients with Y537S *ESR1* mutation might not benefit from everolimus^5,6^. However, these results need further confirmation. We were unable to validate them in our study because our cohort included only two patients with the Y537S *ESR1* mutation.

*PIK3CA* and *ESR1* mutations were previously reported to be related to worse outcome in patients with ER + metastatic breast cancer^6,19^. We were unable to confirm this in our small study with more heavily pre-treated patients. Furthermore, in patients who progressed on non-steroidal aromatase inhibitors, possibly harboring *ESR1* mutations, a SERD might have been a better endocrine partner than exemestane to combine with everolimus.

In our study, the outcome was independent of disease burden, treatment history, and previous treatment with CDK 4/6 inhibitors, whereas, surprisingly, prognosis in younger patients was worse. The subgroup analysis of elderly patients in the BOLERO-2 pilot trial reports that everolimus efficacy is independent of age: Patients older than 65 derive the same magnitude of benefit as younger patients when treatment with everolimus is compared to treatment with exemestane alone^20^.

And, lastly, we acknowledge that having a larger dataset or a different cohort would provide the opportunity to further validate our data.

In the PEARL study, the absence of ^18^F-FDG-PET/CT metabolic response and persistent ctDNA detection after only 14 days of treatment with the EXE-EVE combination is associated with a lower probability of benefit for patients with ER + HER2−, non-steroidal aromatase inhibitor resistant metastatic breast cancer. Independent validation of these results is needed.

While our trial is unlikely to be practice changing, it has the merit to open a new research track that deserves to be actively pursued, given the exponential development of expensive and toxic anticancer therapies. Future trials using improved ctDNA assays and more advanced PET imaging approaches are needed to identify patients who can be spared from ineffective and potentially toxic treatments.

## Methods

### Patient eligibility, study design and treatment

Patients enrolled in this trial were postmenopausal women who were diagnosed with estrogen-receptor-positive (ER + ), HER2−negative breast cancer, refractory to non-steroidal aromatase inhibitors, and eligible for EXE + EVE treatment according to the investigator’s assessment. Other previous endocrine treatments alone or in combination with CDK 4/6 inhibitors and prior chemotherapy for advanced disease were allowed.

Patients were required to have at least one ^18^F-FDG-PET/CT evaluable lesion at baseline. This meant having a marked accumulation of ^18^F-FDG, at least 1.5-fold greater than standard uptake value (SUV) mean + 2 SDs in a 3 cm spherical region of interest (ROI) in a normal right lobe of the liver; if the liver was abnormal, the target lesion should have had uptake >2.0 × SUV mean + 2 SDs of blood pool in 1 cm-diameter ROI in the descending thoracic aorta^21^.

Patients were treated with everolimus 10 mg daily and exemestane 25 mg daily until disease progression or unacceptable adverse events. Treatment interruptions or dose reductions to 5 mg daily of everolimus were allowed for toxicity.

Efficacy was evaluated using classical radiological assessments (CT or MRI) every 12 weeks, applying RECIST 1.1 criteria. ^18^F-FDG-PET/CT was performed at two-time points, namely at baseline and at D14 after treatment initiation, the so-called early ^18^F-FDG-PET/CT.

The trial was approved by the institutional review boards and ethical committees of each participating center, and it was registered in the European Union Drug Regulating Authorities Clinical Trials Database (EudraCT number 2012-004860-22). Written informed consent was obtained from all patients before enrollment. The study was conducted in accordance with the principles of Good Clinical Practice and the provisions of the Declaration of Helsinki and applicable local regulations.

### Objectives

The primary objective of the trial was to evaluate whether the early metabolic response is associated with PFS. All ^18^F-FDG-PET/CT images were evaluated by two nuclear medicine specialists blinded to the clinical data. A patient was considered to be a “responder” when a uniform SUVmax reduction of more than 25% was seen in all lesions (so called consistent patient-based response)^9^. All cases not fulfilling this criterion were classified as “non-responders”.

Considering previous data in the literature about breast cancer patients treated with targeted therapies^12^, we retrospectively decided to assess a lower cut-off than initially defined in the protocol to define metabolic non-response. We thought it would be worthwhile to test this because it was potentially more adapted to distinguish responding patients from the non-responding ones in this particular population. Consequently, we performed a post-hoc analysis defining as responders those patients who showed a uniform reduction of 15% of all lesions.

The aim of the translational research in our study was to explore whether ctDNA detection at baseline or D14 or ctDNA changes between baseline and D14 were associated with PFS. Additionally, we aimed to explore whether combining ^18^F-FDG-PET/CT and ctDNA analysis could improve our ability to identify early on patients who will not benefit from EXE + EVE. Finally, the ctDNA analyses aimed to characterize genomic alterations in ctDNA that emerge at D14 and at progression.

### Imaging procedures and analysis

Whole-body ^18^F-FDG-PET/CT was performed in 5 different Belgian PET centers, all EARL-accredited (earl.eanm.org). The protocol allowed a maximum of 7 days between baseline FDG imaging and treatment start. The baseline ^18^F-FDG-PET/CT-scan and the subsequent scan had to be performed on the same machine.

Before FDG injection, patients were required to have fasted for at least 6 h and to have blood glucose levels <200 mg/dL. The minimum injected activity was 3.7 MBq/kg with a maximum activity of 370 MBq, and the maximum difference allowed between the baseline and early scan was 25%. For all scans, the uptake time was 60–70 min, with no more than a 10 min difference between baseline and subsequent scan.

Since data on ^18^F-FDG-PET/CT in patients treated with everolimus were scarce, we first conducted a pilot phase (*N* = 27, early ^18^F-FDG-PET/CT realized 14 and 28 days respectively after treatment initiation). Metabolic non-response was highly concordant between the two time points. To reduce treatment exposure, we chose D14 for the second phase (*n* = 20).

Early ^18^F-FDG-PET/CT had to be scheduled on D14 (up to D17), with D1 defined as the first day of treatment administration. Early ^18^F-FDG-PET/CT results were blinded to treating oncologists, except in the case of life-threatening progression. An imaging core lab (Orilab, Institut Jules Bordet, Brussels) assessed the image quality and compliance with the imaging guidelines.

The main metabolic response criteria used in this prospective study followed the Consist method with a threshold of 25% (threshold inspired by the EORTC cut-off). Responding patients (R) were those showing consistent homogenous response in all target lesions with a decrease in tracer uptake (SUVmax) of at least 25%, and an absence of new lesions (Class 1 as described below). All other patients were considered to be non-responders (NR)-Classes 2–4 described below.

The Consists criteria, so called dominance method uses the patient-based classification of metabolic response within 4 classes: Class 1 when all target lesions do respond (reduction of SUVmax seen in all lesions), Class 2 describes a mixed response with majority of whole-body tumor load that responds, Class 3 describes a mixed response with majority of whole-body tumor load that does not respond and finally Class 4 describes the situation when all target lesions do not respond or a progressive lesion occurs (i.e. increase of SUVmax in a known lesion or appearance of a new FDG positive lesion)^9^.

Secondly, we used Percist response assessment to evaluate metabolic response: a maximum of 10 target lesions (maximum of 2 per organ) were be identified. If more than 2 target lesions within one organ are available only 2 lesions with the highest SUV are defined as target lesions. SUVpeak at baseline and during the treatment are calculated for all target lesions. Significant lesion response is defined as a relative decrease of SUVpeak of more than 30% therefore the patient will be classified as followed: metabolic responder (MR) when the difference between the SUVpeak of the hottest lesion at baseline and during treatment is more than 30% or metabolic non-responder (MNR) when the definition of MR is not fulfilled^21^.

As described above (Objectives section), a post-hoc analysis using a 15% cut-off was performed.

The selection of the target lesion was defined according to PERCIST (size ≥1.5 cm and uptake above the PERCIST threshold)^21^.

### Blood sample collection, ctDNA isolation, and analysis

10 ml of whole blood was collected in EDTA tubes at the following time points: baseline, D14 (at the same time as the early ^18^F-FDG-PET/CT), at all RECIST 1.1 evaluations, and at disease progression. Only plasma samples taken at baseline, D14, and at progression were analyzed. Plasma was prepared within a maximum of 30 min after blood collection by double centrifugation: first at 850 g at 4 °C for 10 min, and at full speed (18.000–20.000 g) for 10 more minutes thereafter. Plasma samples were stored at −70 °C until shipped to the central laboratory at Institut Jules Bordet in Brussels.

Cell-free DNA (cfDNA) was extracted from blood using the Qiagen DNA Blood Mini Kit (Qiagen, Valencia, USA). DNA quantity was measured using the Qubit 2.0 Fluorometer (Thermo Fisher Scientific, Waltham, USA).

#### ctDNA analysis

Plasma ctDNA was sequenced to identify hotspot mutations in 40 cancer-specific genes (Supplementary Table 1) with coverage of 15000× using the commercially available next-generation sequencing panel. For variants classification, we followed the Compermed method^22^.

The targeted sequencing libraries were generated using the Ion AmpliSeq Library kit 2.0 according to the manufacturer’s instructions (Thermo Fisher Scientific, Waltham, USA). The starting material consisted of 10 ng DNA from plasma samples per pool of amplification. The primers used for amplification were partially digested by the Pfu enzyme. The product of digestion was then ligated with corresponding barcoded adapters and purified using Ampure Beads (Beckman Coulter Inc., Indianapolis, USA). The product of purification was amplified for 5 more cycles and subsequently re-purified using Ampure Beads to generate the library sample. The quality of the libraries was assessed using the Qubit dsDNA HS Assay kit (Thermo Fisher Scientific, Waltham, USA). Ten pmol/L of each library was loaded into the IonChef system (Thermo Fisher Scientific, Waltham, USA) for the emulsion polymerase chain reaction. Libraries were then loaded into the sequencing chip that was placed in either the Personal Genome Machine, the Proton, or the 5XL device (Thermo Fisher Scientific, Waltham, USA) depending on the required throughput.

Primary processing of next-generation sequencing data and identification of putative somatic mutations: The data generated were first aligned to the human reference sequence and annotated using the Consensus Coding DNA Sequences, RefSeq, and Ensembl databases. NGS data were then analyzed using the Torrent Suite Software (Thermo Fisher Scientific, Waltham, USA). Next, somatic mutations were identified with the Variant Caller 4.0 software (Thermo Fisher Scientific, Waltham, USA) using the somatic high stringency parameters to ensure sufficient coverage of the analyzed bases and to exclude mapping and sequencing errors. Genetic aberration analysis was focused on single-base substitutions, small insertions, and deletions. Candidate somatic alterations were further filtered based on: coverage of >100; a forward-reverse ratio of 10%, 90%; the exclusion of intronic and silent changes; and the retention of mutations resulting in missense mutations, nonsense mutations, frameshifts, or splice site alterations in the protein-coding region. A manual visual inspection step was used to further remove artefactual changes.

### Statistical plan

We assumed that the ^18^F-FDG-PET/CT responders would have a PFS similar to that of the patients treated with EXE-EVE in the BOLERO-2 trial (10.6 months) and that non-responders would have a PFS similar to that of patients treated with exemestane alone (4.1 months). Under exponential survival, this translated to an HR of 0.36. To show an increase in 6-month PFS from 37% for non-responders to 70% for responders (corresponding to an HR of 0.36) with 90% power and a two-sided significance level of 0.05 using a Log rank test and, considering the results of the pilot phase (see below), a total of 42 PFS events were required for the analysis. To answer the primary objective of the trial, a total of 46 evaluable patients were needed. PFS was defined as the time interval between the date of the second PET and the date of progression or death.

A Cox regression model was applied to assess the effect of ^18^F-FDG-PET/CT metabolic response rate and the effect of somatic mutations detected in ctDNA on PFS.

### Reporting summary

Further information on research design is available in the Nature Research Reporting Summary linked to this article.

## Supplementary information

Supplementary Information

Reporting Summary

## Data Availability

Single nucleotide variants (SNVs) identified in each individual patient, variant allele frequency (VAF) and the number of total and mutated copies/ml of plasma at three different time-points are available in Supplementary Table 2. Raw data that support the findings of ctDNA targeted gene sequencing included in this manuscript are available on EGA under the identifier EGAS00001005523. All other data is available from the corresponding author upon reasonable request.

## Code availability

All custom scripts used in this study are available upon request. The statistical analyses were performed with SAS 9.4 (SAS Institute Inc., Cary, NC, USA) and R 4.0.2.
