# Author Correction: FDG positron emission tomography imaging and ctDNA detection as an early dynamic biomarker of everolimus efficacy in advanced luminal breast cancer

**DOI:** 10.1038/s41523-022-00409-x

**Published:** 2022-03-16

**Authors:** Andrea Gombos, David Venet, Lieveke Ameye, Peter Vuylsteke, Patrick Neven, Vincent Richard, Francois P. Duhoux, Jean-Francois Laes, Françoise Rothe, Christos Sotiriou, Marianne Paesmans, Ahmad Awada, Thomas Guiot, Patrick Flamen, Martine Piccart-Gebhart, Michail Ignatiadis, Géraldine Gebhart

**Affiliations:** 1 Medical Oncology Department, Institut Jules Bordet and Université Libre de Bruxelles (ULB), Brussels, Belgium; 2 Université Libre de Bruxelles, Brussels, Belgium; 3 Breast Cancer Translational Research Laboratory, Institut Jules Bordet and Université Libre de Bruxelles (ULB), Brussels, Belgium; 4 Statistical Department, Institut Jules Bordet, Université Libre de Bruxelles (ULB), Brussels, Belgium; 5 Medical Oncology, UCLouvain, CHU Namur Site Sainte-Elisabeth, Namur, Belgium; 6 University of Botswana, Private Bag UB 0022, Gabarone, Botswana; 7 Multidisciplinary Breast Center and Department of Gynecology and Obstetrics, UZ Leuven, KU Leuven, Leuven, Belgium; 8 Medical Oncology CHU Ambroise Paré, Mons, Belgium; 9 Medical Oncology Cliniques Universitaires Saint-Luc, Brussels, Belgium; 10 OncoDNA, Gosselies, Belgium; 11 Nuclear Medicine, Institut Jules Bordet, Brussels, Belgium

**Keywords:** Breast cancer, Breast cancer

Correction to: *npj Breast Cancer* 10.1038/s41523-021-00331-8, published online 21 September 2021

The Funding information section was missing from this article and should have read “The authors would like to thank to the Fonds de la Recherche Scientifique - FNRS for providing funding for this study through the PDR T025918F grant”. The original article has been corrected.

