# Supplementary material for: FDG positron emission tomography imaging and ctDNA detection as an early dynamic biomarker of everolimus efficacy in advanced luminal breast cancer
Source: NPJ Breast Cancer. 2021 Sep 21;7:125. doi: 10.1038/s41523-021-00331-8 (PMC8455671; doi:10.1038/s41523-021-00331-8)
Supplement: Supplementary file 1 — Supplementary Information [file 41523_2021_331_MOESM1_ESM.pdf]

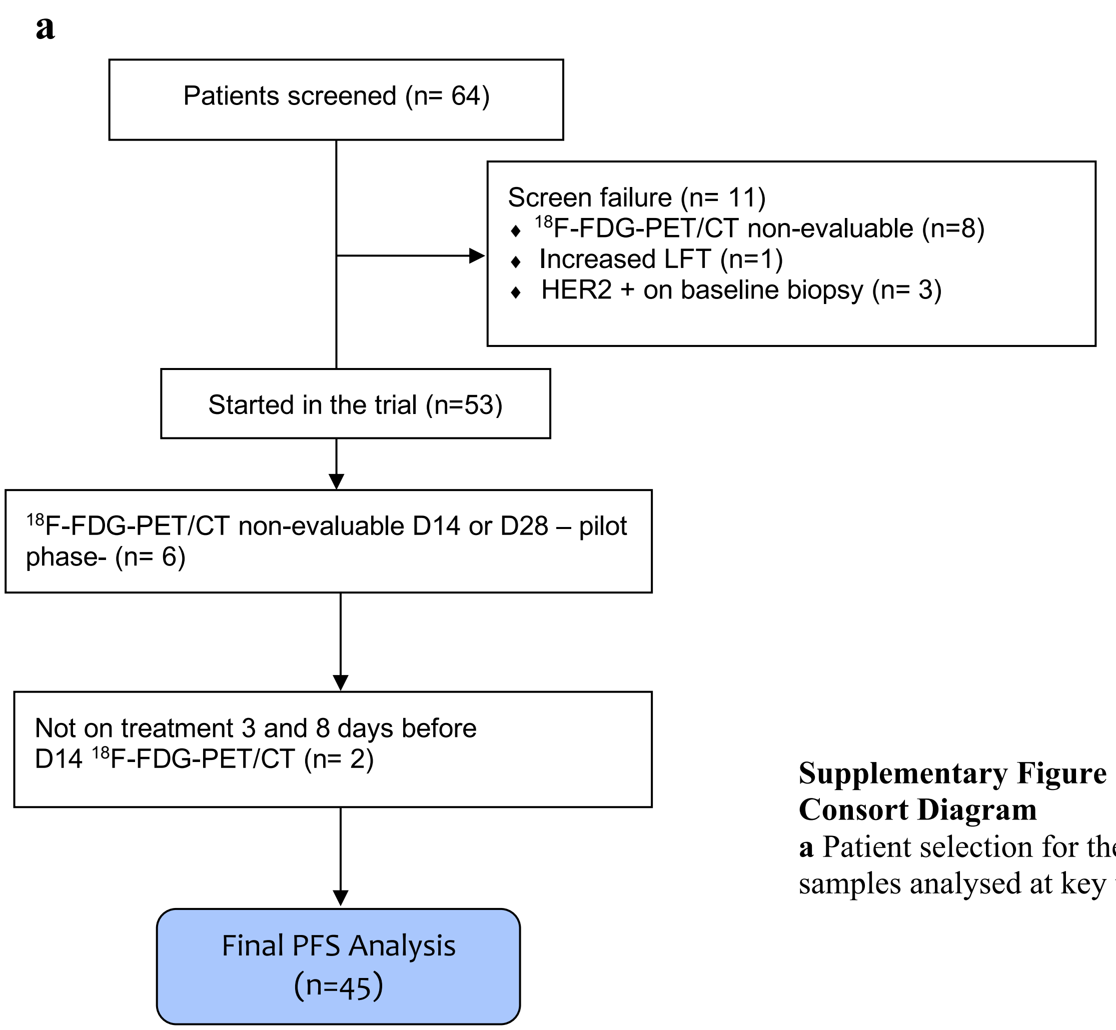

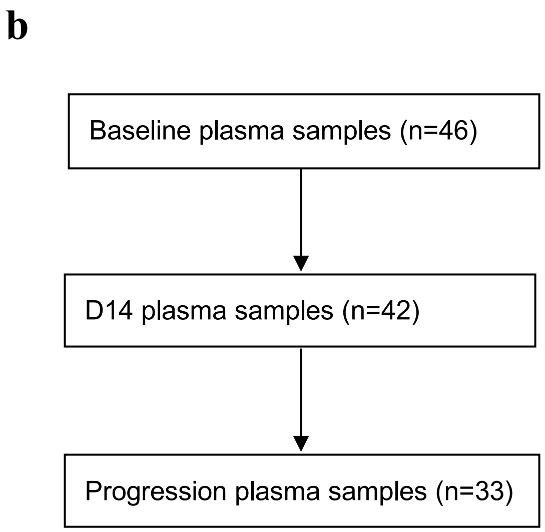

Supplementary Figure 1
Consort Diagram

a Patient selection for the main ^{18}F-FDG-PET/CT analysis; **b** Number of sequential plasma samples analysed at key timepoints. Four samples were missing on D14 and 13 at progression

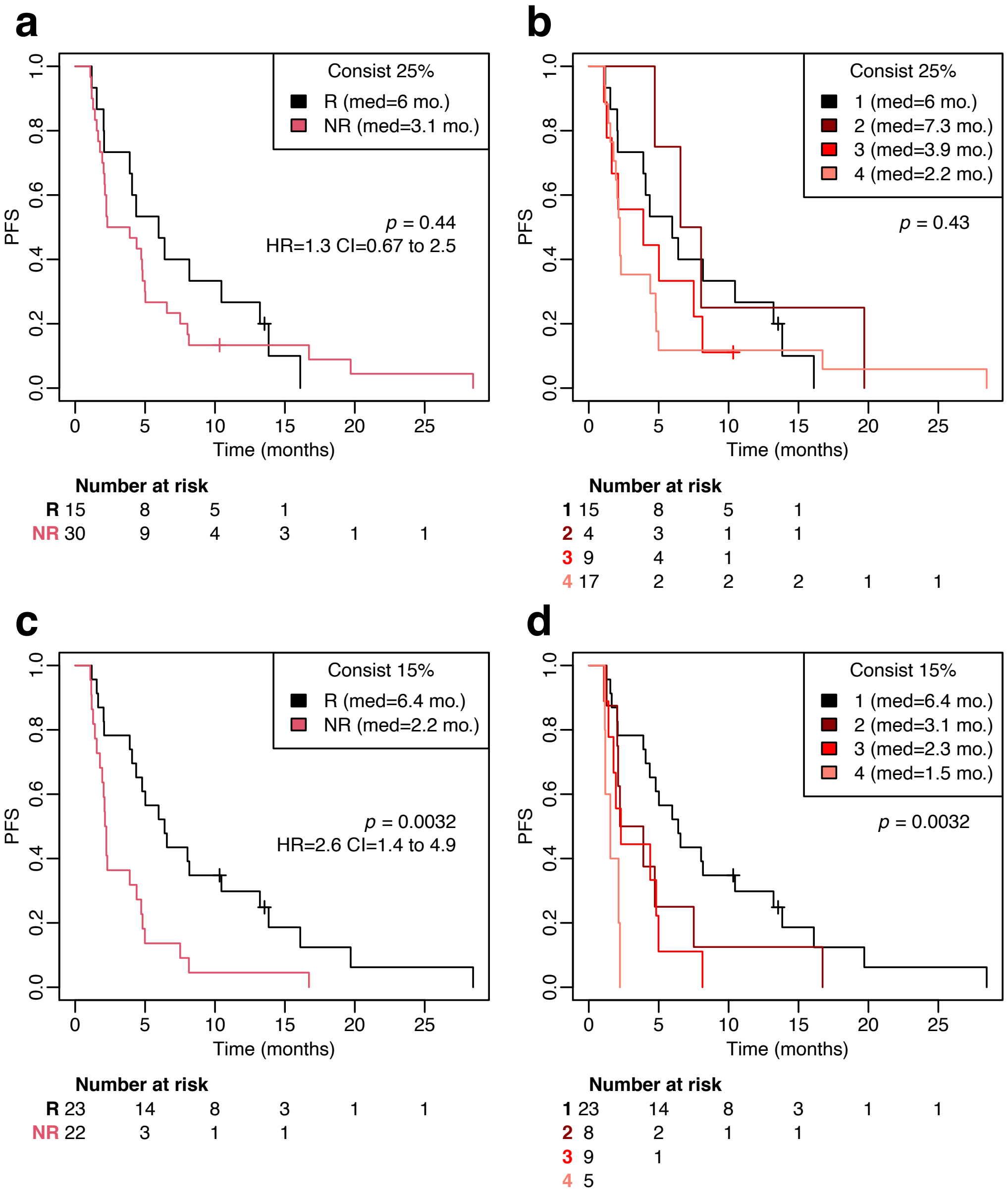

Supplementary Figure 2

Kaplan-Meier plots of progression free survival (PFS) according to ^{18}F-FDG-PET/CT evaluation 14 days after the start of treatment with exemestane-everolimus.

a PET evaluation using two classes (response R, non response NR): Response is considered as ≥ 25% homogenous decrease in maximum standardized uptake value (SUVmax) in all target lesions (R), failure to fulfil this criterion is considered non-response (NR)

b PET evaluation using 4 classes and 25% cut-off: 1 – no metabolic non-responsive lesion, 2 – minority of lesions are non-responders among the whole body target lesions, 3 - majority of lesions are non-responders among the whole body target lesions, 4 – none of the target lesions shows a response

c PET evaluation using two classes (response R, non response NR): Response is considered as ≥ 15% homogenous decrease in maximum standardized uptake value (SUVmax) in all target lesions (R), failure to fulfil this criterion is considered non-response (NR)

d PET evaluation using 4 classes and 15% cut off: 1 – no metabolic non-responsive lesion, 2 – minority of lesions are non-responders among the whole body target lesions, 3 - majority of lesions are non-responders among the whole body target lesions, 4 – none of the target lesions shows a response

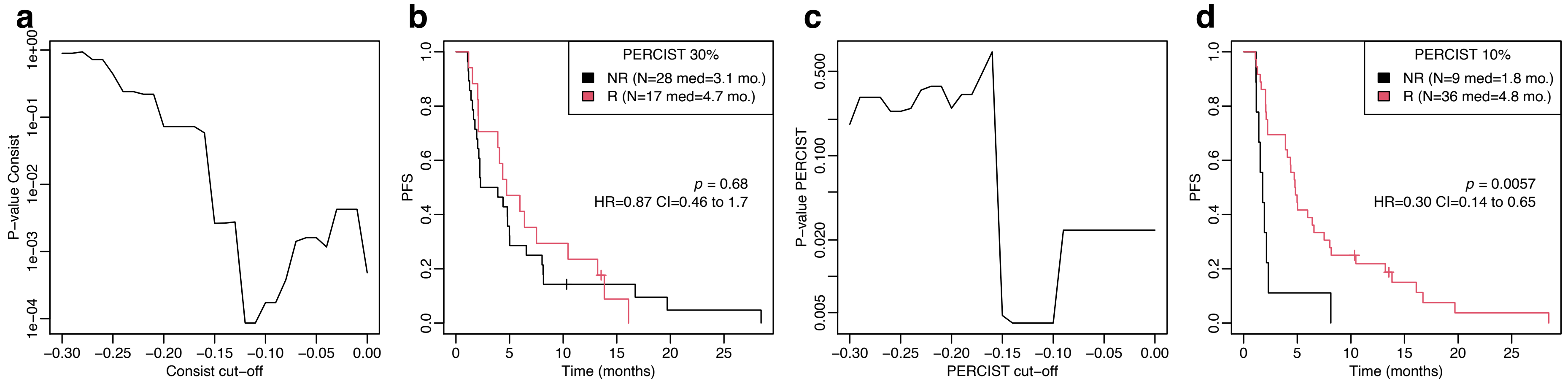

Supplementary Figure 3

Ability of differently defined metabolic responses to predict PFS. **a** Evolution of the p-value of the link between PFS and metabolic response, with varying Consist cut-offs. **b** PFS according to PERCIST (Positron Emission Tomography –PET- Response Criteria in Solid Tumors) with the standard 30% threshold. **c** Effect of the cut-offs on PERCIST. **d** PERSIST *vs.* PFS using the 10% cut-off.

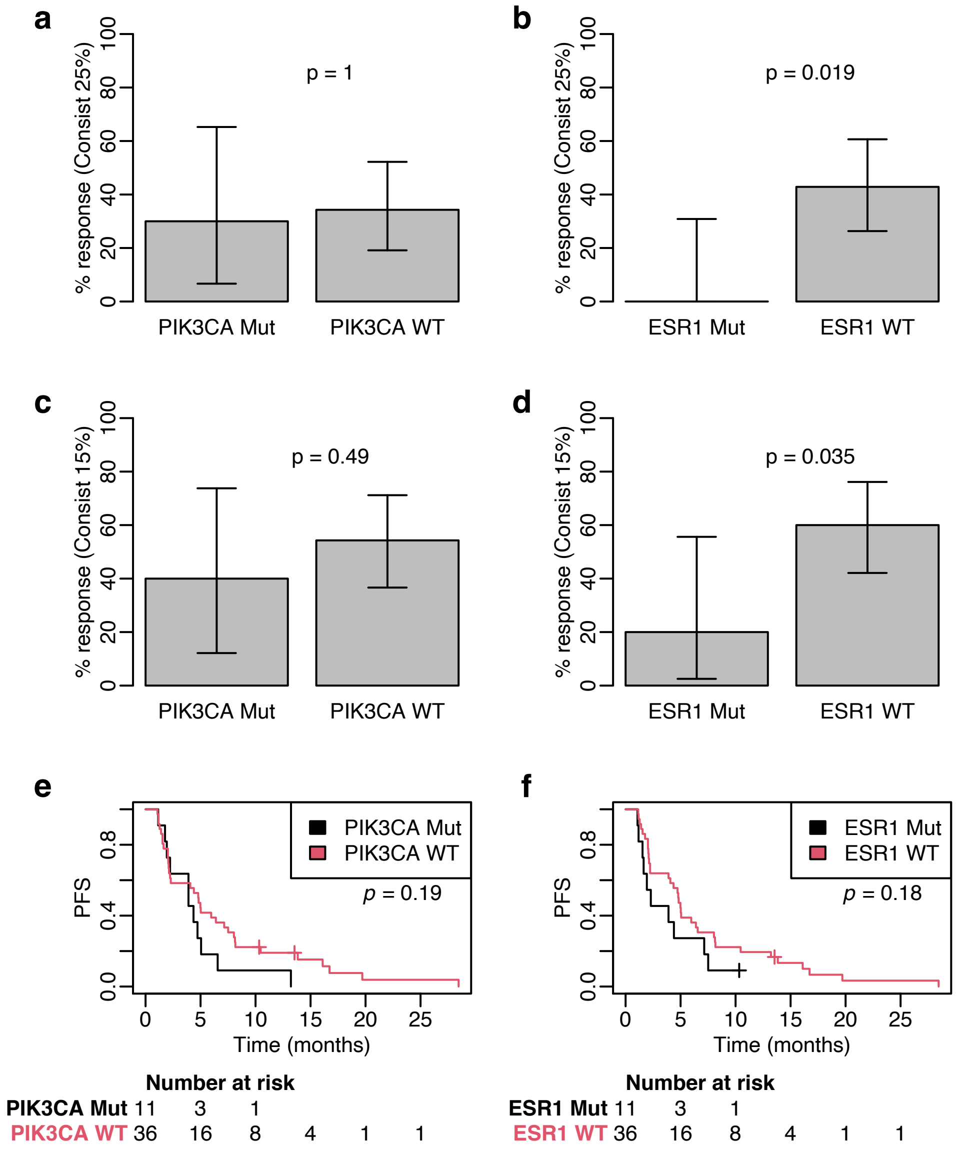

Supplementary Figure 4
Association between ctDNA mutation detection in *PIK3CA* and *ESR1* genes at baseline and either

^{18}F-FDG-PET/CT response according to Consist criteria using a 25% cut-off **(a, b) or**

^{18}F-FDG-PET/CT response according to Consist criteria using a 15% cut-off **(c, d)** or

Progression Free Survival, PFS **(e, f)**

In panels a-d data represented are mean values. Error bars represent 95% confidence intervals (obtained from the binomial test in R). “p” denotes p values.

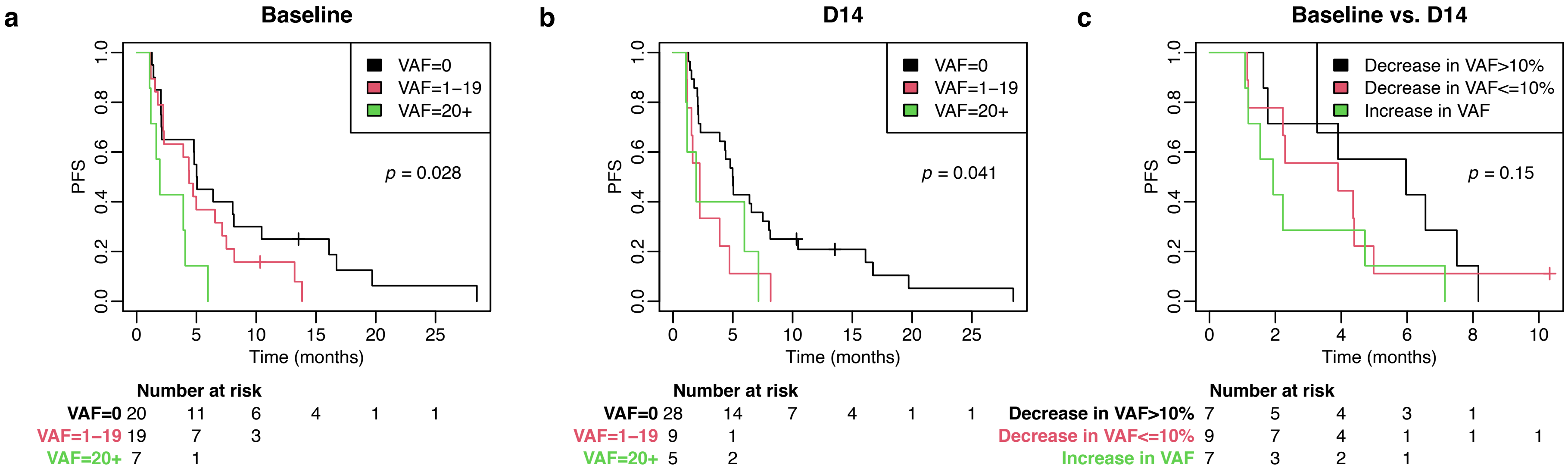

Supplementary Figure 5

Prognostic value of Variant allele frequency (VAF) of SNVs (single nucleotide variants) detected by targeted gene sequencing in plasma cell free DNA at different time-points. VAF is expressed in %.

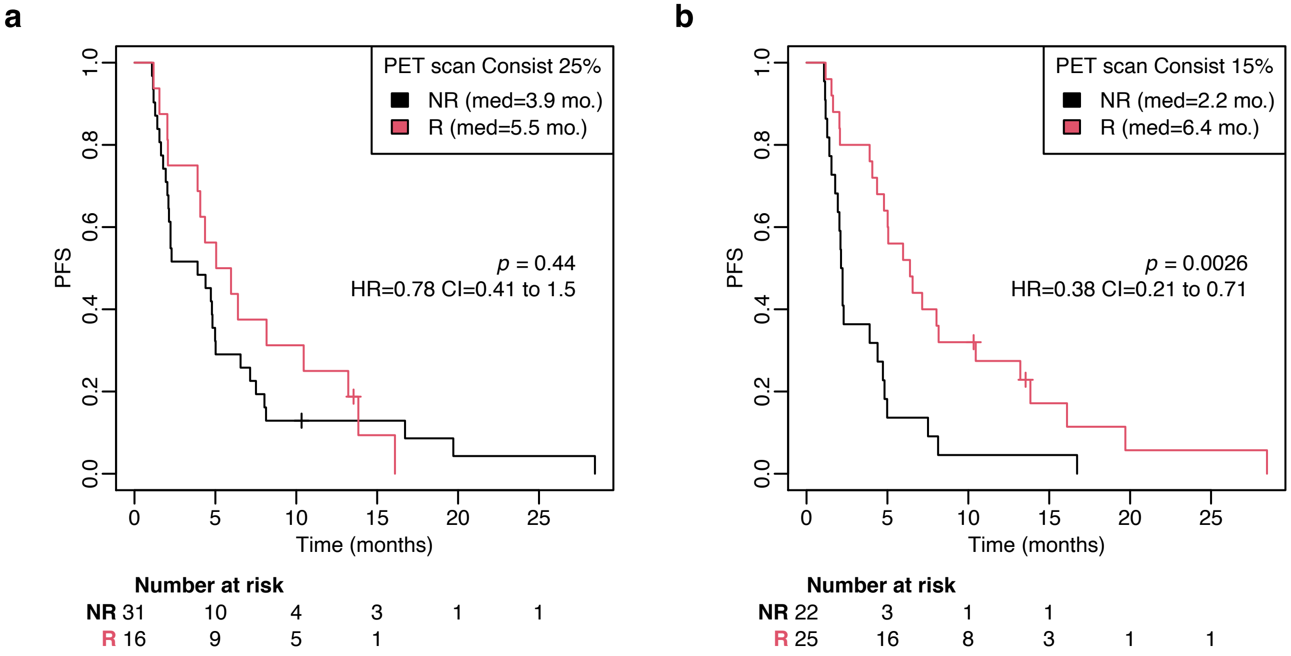

Supplementary Figure 6 (refers to "data not shown" on page 8 of the manuscript)
Kaplan-Meier plots of progression free survival (PFS) according to ^{18}F-FDG-PET/CT evaluation 14 days after the start of treatment with exemestane-everolimus in the ITT population (n=47)

a. Consist 15%: Response is considered as ≥ 15% homogenous decrease in maximum standardized uptake value (SUVmax) in all target lesions (R), Failure to fulfil this criterion is considered non-response (NR)

b. Consist 25%: Response is considered as ≥ 25% homogenous decrease in maximum standardized uptake value (SUVmax) in all target lesions (R), Failure to fulfil this criterion is considered non-response (NR)

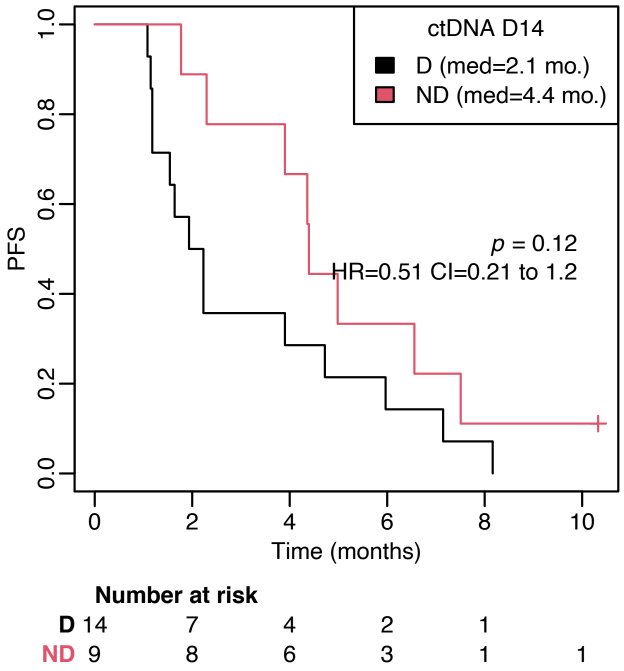

Supplementary Figure 7
Kaplan-Meier plot of progression free survival (PFS) according to ctDNA detection 14 days after the start of treatment with exemestane-everolimus in patients in whom ctDNA was detected at baseline and had available sample on D14 (n=23)

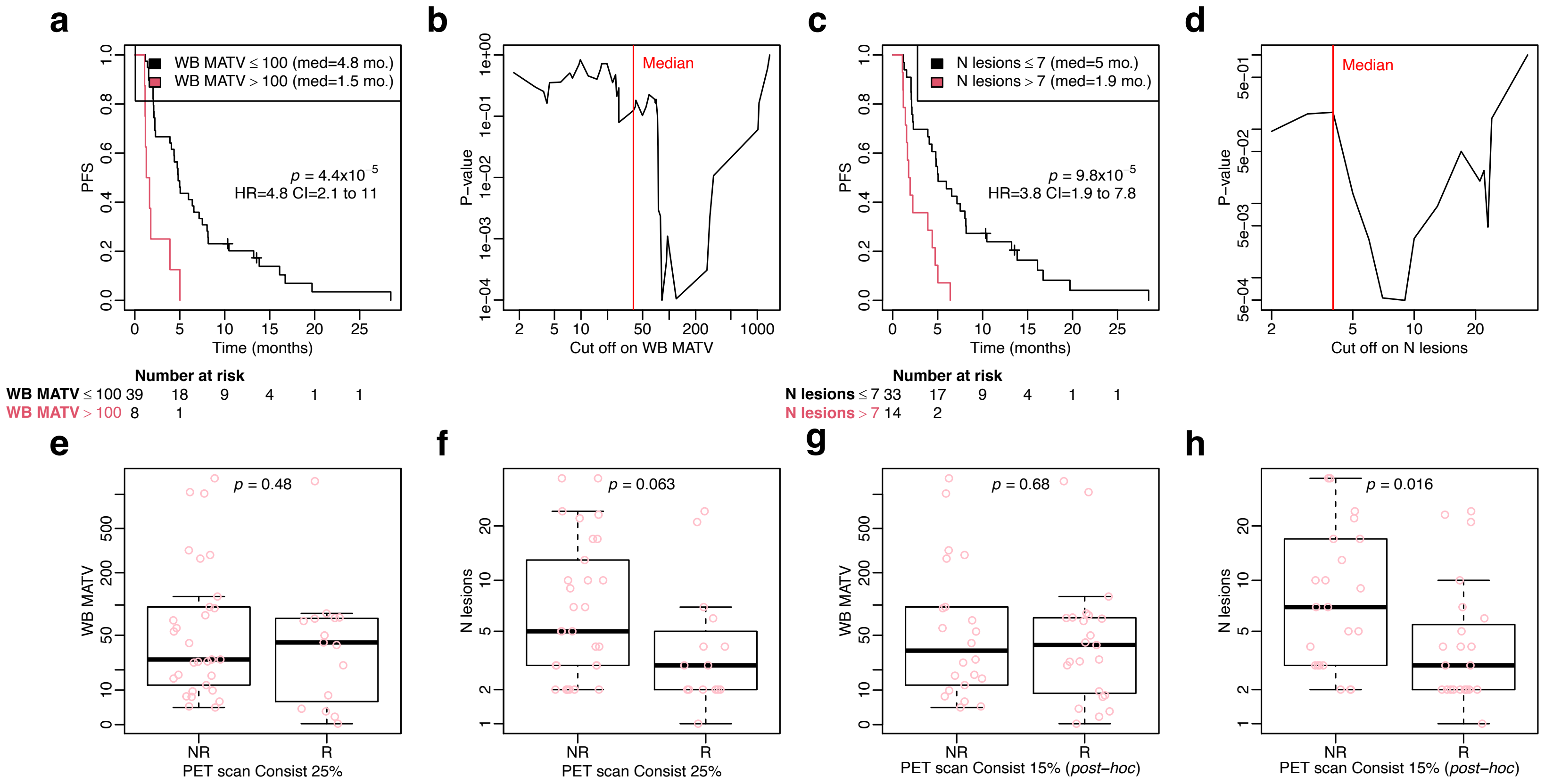

Supplementary Figure 8

Kaplan-Meier plots of progression free survival (PFS) and ^{18}F-FDG-PET/CT response according to Metabolically Active Tumor Volume (MATV) and number of metabolically active metastatic lesions assessed on baseline ^{18}F-FDG-PET/CT. a PFS in patients with MATV > 100 cm^3 compared to PFS of patients with MATV ≤100 cm^3. **b** Evolution of p value with varying MATV cut-offs. **c** PFS in patients with > 7 metastatic lesions compared to PFS of patients with ≤7 metastatic lesions. **d** Evolution of p value with varying cut-off for the number of metastatic lesions. **e** Association between ^{18}F-FDG-PET/CT response (according to Consist criteria using a 25% cut-off) and MATV**. f** Association between ^{18}F-FDG-PET/CT response (according to Consist criteria using a 25% cut-off) and the number of metastatic lesions**. g** Association between ^{18}F-FDG-PET/CT response (according to Consist criteria using a 15% cut-off) and MATV**. h** Association between ^{18}F-FDG-PET/CT response (according to Consist criteria using a 15% cut-off) and MATV. Box plots are standard R boxplots, in that the boxes represent interquartile range (IQR) while the whiskers extend to the furthest points that are closer than 1.5IQR from the box.

AKT1	ALK	AR	BRAF	BTK	CTNNB1	DDR2	EGFR
ERBB2	ESR1	EZH2	FBXW7	FGFR1	FGFR2	FGFR3	FOXL2
GNA11	GNAQ	GNAS	HRAS	IDH1	IDH2	JAK2	JAK3
KIT	KRAS	MAP2K1	MAP2K2	MET	MPL	MTOR	NPM1
NRAS	PDGFRA	PIK3CA	PTEN	RAF1	RET	ROS1	TP53

Supplementary Table 1
Targeted gene sequencing panel used for sequencing of plasma samples able to identify single nucleotide variants (SNVs) from 40 genes

Patient	cfDNA baseline (genomes/ml)	cfDNA D14 (genomes/ml)	cfDNA progression (genomes/ml)	Nmut	Gene	Chr	Start	End	cDNA Variant	Amino Acid Variant	Biological Impact	VAF baseline (%)	mutated cfDNA baseline (genomes/ml)	VAF D14 (%)	mutated cfDNA D14 (genomes/ml)	VAF progression (%)	mutated cfDNA progression (genomes/ml)	Gene 2	Chr 2	Start 2	End 2	cDNA Variant 2	Amino Acid Variant 2	Biological Impact 2	VAF baseline (%) 2	mutated cfDNA baseline (genomes/ml) 2	VAF D14 (%) 2	mutated cfDNA D14 (genomes/ml) 2	VAF progression (%) 2	mutated cfDNA progression (genomes/ml) 2
**2**	2677575.758	235454.545	712121.2121	2	ESR1	chr6	151977826	152450754	c.1613A>G	p.D538G	pathogenic	21	562290.9091	8	18836.36364	13	92575.75758	PIK3CA	chr3	178865902	178957881	c.1624G>A	p.E542K	pathogenic	24	642618.1818	10	23545.45455	15	106818.1818
**3**	31212.12121	48484.8485	32121.21212	1	ESR1	chr6	151977826	152450754	c.1613A>G	p.D538G	pathogenic	6	1872.727273	2	969.6969697	2	642.4242424													
**5**	33333.33333	78484.8485	59696.9697	0																										
**7**	103636.3636	149696.97	83333.33333	2	ESR1	chr6	151977826	152450754	c.1613A>G	p.D538G	pathogenic	33	34200	42	62872.72727	16	13333.33333	PIK3CA	chr3	178865902	178957881	c.1633G>A	p.E545K	pathogenic	36	37309.09091	37	55387.87879	21	17500
**9**	91212.12121	41818.1818		2	ESR1	chr6	151977826	152450754	c.1609T>A	p.Y537N	pathogenic	7	6384.848485	0	0			TP53	chr17	7565097	7590856	c.1146del	p.K382Nfs*40	pathogenic	16	14593.93939	0	0		
**12**	59090.90909	47272.7273	67878.78788	2	PIK3CA	chr3	178865902	178957881	c.1633G>C	p.E545Q	pathogenic	0	0	12	5672.727273	9	6109.090909	TP53	chr17	7565097	7590856	c.625A>T	p.R209*	pathogenic	7	4136.363636	15	7090.909091	24	16290.90909
**20**	56060.60606		137575.7576	2	PTEN	chr10	89622870	89731687	c.275A>T	p.D92V	pathogenic	8	4484.848485			9	12381.81818	TP53	chr17	7565097	7590856	c.22C>T	p.P8S	vus	45	25227.27273			41	56406.06061
**27**	42424.24242	63939.3939	120606.0606	1	PIK3CA	chr3	178865902	178957881	c.1035T>A	p.N345K	pathogenic	4	1696.969697	8	5115.151515	15	18090.90909													
**28**	87272.72727		58787.87879	1	AKT1	chr14	105235686	105262088	c.49G>A	p.E17K	pathogenic	8	6981.818182			6	3527.272727													
**29**	54545.45455	77575.7576	67575.75758	0																										
**30**	43636.36364	40909.0909	144545.4545	1	PIK3CA	chr3	178865902	178957881	c.1035T>A	p.N345K	pathogenic	19	8290.909091	0	0	33	47700													
**33**	90000	65454.5455	118484.8485	0																										
**34**	49090.90909	101515.152	44242.42424	0																										
**35**	119393.9394	174545.455	756666.6667	1	ERBB2	chr17	37844167	37886679	c.2313_2324dup	p.Y772_A775dup	pathogenic	10	11939.39394	4	6981.818182	6	45400													
**39**	45454.54545	54545.4545	60606.06061	0																										
**42**	478787.8788	300606.061	166969.697	0																										
**44**	3253030.303	2636060.61		1	TP53	chr17	7565097	7590856	c.343del	p.H115Ifs*8	pathogenic	64	2081939.394	78	2056127.273															
**45**	60000	103939.394	193333.3333	0																										
**46**	62424.24242	58181.8182	54545.45455	1	ESR1	chr6	151977826	152450754	c.1613A>G	p.D538G	pathogenic	4	2496.969697	0	0	1	545.4545455													
**47**	40000	50000	158181.8182	1	ESR1	chr6	151977826	152450754	c.1609T>A	p.Y537N	pathogenic	3	1200	0	0	1	1581.818182													
**49**	1883636.364	332121.212		1	ESR1	chr6	151977826	152450754	c.1613A>G	p.D538G	pathogenic	40	753454.5455	1	3321.212121															
**50**	62121.21212	156969.697	150606.0606	1	PIK3CA	chr3	178865902	178957881	c.1633G>A	p.E545K	pathogenic	4	2484.848485	1	1569.69697	4	6024.242424													
**52**	111818.1818	558484.848	476060.6061	0																										
**51**	46363.63636	75151.5152		0																										
**53**	119393.9394	106060.606	113636.3636	1	PIK3CA	chr3	178865902	178957881	c.3140A>T	p.H1047L	pathogenic	2	2387.878788	0	0	2	2272.727273													
**55**	101515.1515	110303.03	123030.303	0																										
**56**	109393.9394	87272.7273		1	AKT1	chr14	105235686	105262088	c.49G>A	p.E17K	pathogenic	3	3281.818182	0	0															
**59**	231818.1818	156969.697		0																										
**62**	226969.697			0																										
**61**	63939.39394	432424.242		1	PIK3CA	chr3	178865902	178957881	c.1633G>A	p.E545K	pathogenic	3	1918.181818	0	0															
**64**		179090.909		0																										
**11**	83636.36364	86060.6061	889696.9697	1	AKT1	chr14	105235686	105262088	c.49G>A	p.E17K	pathogenic	12	10036.36364	1	860.6060606	38	338084.8485													
**14**	50000		48181.81818	0																										
**17**	61515.15152	175454.545	94545.45455	1	EZH2	chr7	148504475	148581413	c.148508717-6C>T	-	vus	56	34448.48485	40	70181.81818	41	38763.63636													
**19**	82121.21212		151212.1212	1	PIK3CA	chr3	178865902	178957881	c.1258T>C	p.C420R	pathogenic	4	3284.848485			14	21169.69697													
**22**		293030.303	162121.2121	1	PIK3CA	chr3	178865902	178957881	c.1340_1366del	p.P447_L455del	pathogenic	0		0	0	2	3242.424242													
**24**	94545.45455	76666.6667	80909.09091	0																										
**26**	56666.66667	88484.8485	327878.7879	1	ESR1	chr6	151977826	152450754	c.1613A>G	p.D538G	pathogenic	14	7933.333333	19	16812.12121	33	108200													
**32**	35454.54545	175757.576	78181.81818	0																										
**40**	52121.21212	114545.455	52424.24242	0																										
**41**	57575.75758	46060.6061		0																										
**48**	113636.3636	87272.7273		2	PIK3CA	chr3	178865902	178957881	c.3140A>G	p.H1047R	pathogenic	16	18181.81818	0	0			TP53	chr17	7565097	7590856	c.839G>C	p.R280T	pathogenic	13	14772.72727	0	0		
**54**	40000	98484.8485		0																										
**57**	53636.36364	285454.545	84848.48485	0																										
**58**	25757.57576	52727.2727		1	ESR1	chr6	151977826	152450754	c.1613A>G	p.D538G	pathogenic	3	772.7272727	0	0															
**60**	182121.2121	182424.242	2227878.788	2	ESR1	chr6	151977826	152450754	c.1610A>C	p.Y537S	pathogenic	21	38245.45455	25	45606.06061	38	846593.9394	PTEN	chr10	89622870	89731687	c.416del	p.L139Yfs*8	pathogenic	23	41887.87879	26	47430.30303	36	802036.3636
**63**	86666.66667			2	AKT1	chr14	105235686	105262088	c.49G>A	p.E17K	pathogenic	2	1733.333333	26				ESR1	chr6	151977826	152450754	c.1610A>C	p.Y537S	pathogenic	0	0	21			

Supplementary Table 2

Single nucleotide variants (SNVs) identified in each individual patient, variant allele frequency (VAF) and the number of total and mutated copies/ml of plasma at three different time-point (baseline, D14 and progression).

cfDNA (genomes/ml): total circulating free DNA; Nmut-number of mutations, Chr-chromosome,
