# Supplementary material for: FDG positron emission tomography imaging and ctDNA detection as an early dynamic biomarker of everolimus efficacy in advanced luminal breast cancer
Source: NPJ Breast Cancer. 2021 Sep 21;7:125. doi: 10.1038/s41523-021-00331-8 (PMC8455671; doi:10.1038/s41523-021-00331-8)
Supplement: Supplementary file 2 — Reporting Summary [file 41523_2021_331_MOESM2_ESM.pdf]

nature portfolio

Corresponding author(s): Andrea Gombos, MD
andrea.gombos@bordet.be

Last updated by author(s): Aug 13, 2021

Reporting Summary

Nature Portfolio wishes to improve the reproducibility of the work that we publish. This form provides structure for consistency and transparency in reporting. For further information on Nature Portfolio policies, see our Editorial Policies and the Editorial Policy Checklist.

Statistics

For all statistical analyses, confirm that the following items are present in the figure legend, table legend, main text, or Methods section.

n/a	Confirmed	
☐	☒	The exact sample size (*n*) for each experimental group/condition, given as a discrete number and unit of measurement
☐	☒	A statement on whether measurements were taken from distinct samples or whether the same sample was measured repeatedly
☐	☒	The statistical test(s) used AND whether they are one- or two-sided<br>*Only common tests should be described solely by name; describe more complex techniques in the Methods section.*
☒	☐	A description of all covariates tested
☒	☐	A description of any assumptions or corrections, such as tests of normality and adjustment for multiple comparisons
☐	☒	A full description of the statistical parameters including central tendency (e.g. means) or other basic estimates (e.g. regression coefficient) AND variation (e.g. standard deviation) or associated estimates of uncertainty (e.g. confidence intervals)
☐	☒	For null hypothesis testing, the test statistic (e.g. *F*, *t*, *r*) with confidence intervals, effect sizes, degrees of freedom and *P* value noted<br>*Give P values as exact values whenever suitable.*
☒	☐	For Bayesian analysis, information on the choice of priors and Markov chain Monte Carlo settings
☒	☐	For hierarchical and complex designs, identification of the appropriate level for tests and full reporting of outcomes
☐	☒	Estimates of effect sizes (e.g. Cohen's *d*, Pearson's *r*), indicating how they were calculated

Our web collection on statistics for biologists contains articles on many of the points above.

Software and code

Policy information about availability of computer code

Data collection	No software was used
Data analysis	SAS 9.4 (SAS Institute Inc., Cary, NC, USA) and R 4.0.2.

For manuscripts utilizing custom algorithms or software that are central to the research but not yet described in published literature, software must be made available to editors and reviewers. We strongly encourage code deposition in a community repository (e.g. GitHub). See the Nature Portfolio guidelines for submitting code & software for further information.

Data

Policy information about availability of data

All manuscripts must include a data availability statement. This statement should provide the following information, where applicable:

- Accession codes, unique identifiers, or web links for publicly available datasets
- A description of any restrictions on data availability
- For clinical datasets or third party data, please ensure that the statement adheres to our policy

The data that support the findings of ctDNA targeted gene sequencing included in this manuscript will be charged soon on https://ega-archive.org. All other data is available from the corresponding author upon reasonable request.

Field-specific reporting

Please select the one below that is the best fit for your research. If you are not sure, read the appropriate sections before making your selection.

☒ Life sciences ☐ Behavioural & social sciences ☐ Ecological, evolutionary & environmental sciences

For a reference copy of the document with all sections, see nature.com/documents/nr-reporting-summary-flat.pdf

Life sciences study design

All studies must disclose on these points even when the disclosure is negative.

Sample size	We assumed that the 18F-FDG-PET/CT responders would have a PFS similar to that of the patients treated with exemestane-everolimus in the BOLERO-2 trial (10.6 months) and that non-responders would have a PFS similar to that of patients treated with exemestane alone (4.1 months). Under exponential survival, this translated to a HR of 0.36. To show an increase in 6-month PFS from 37% for non- responders to 70% for responders (corresponding to a HR of 0.36) with 90% power and a two-sided significance level of 0.05 using a Logrank test and, considering the results of the pilot phase (see description in the manuscript), a total of 42 PFS events were required for the analysis. To answer the primary objective of the trial, a total of 46 evaluable patients were needed. PFS was defined as the time interval between the date of the second PET and the date of progression or death.<br>A Cox regression model was applied to assess the effect of 18F-FDG-PET/CT metabolic response rate and the effect of somatic mutations detected in ctDNA on PFS.
Data exclusions	Two patients were excluded from the metabolic response analysis because they stopped everolimus at 3 and 8 days respectively before the D14 18F-FDG-PET/CT was performed.
Replication	This applies mainly for preclinical studies. Regarding 18F-FDG-PET/CT result interpretation: in every patient the response was assessed by two independent and blinded nuclear medicine experts. Consensus reading was organized in case of discordant assessment. In order to ensure image quality, reproducibility and standardization, all participating centers were required to have accreditation through EARL and needed to comply with the requirements of continued quality control as outlined in EARL's accreditation manual (http://earl.eanm.org). Additionally an imaging core lab (Orilab, Institut Jules Bordet, Brussels) assessed the image quality and compliance with the imaging guidelines.
Randomization	Our study is not randomized, it is single arm. Thus, this question is not applicable
Blinding	Blinding is not relevant to our study, patients received standard medication by exemestane-everolimus. Regarding imaging procedures, early 18F-FDG-PET/CT results were blinded to treating oncologists, except in case of life-threatening progression.

Reporting for specific materials, systems and methods

We require information from authors about some types of materials, experimental systems and methods used in many studies. Here, indicate whether each material, system or method listed is relevant to your study. If you are not sure if a list item applies to your research, read the appropriate section before selecting a response.

Materials & experimental systems

n/a	Involved in the study	
☒	☐	Antibodies
☒	☐	Eukaryotic cell lines
☒	☐	Palaeontology and archaeology
☒	☐	Animals and other organisms
☐	☒	Human research participants
☐	☒	Clinical data
☒	☐	Dual use research of concern

Methods

n/a	Involved in the study	
☒	☐	ChIP-seq
☒	☐	Flow cytometry
☒	☐	MRI-based neuroimaging

Human research participants

Policy information about studies involving human research participants

Population characteristics	The patients were all post-menopausal women, mean age at inclusion 57 years (+/- 11 years). They were all diagnosed with ER+/HER2- metastatic breast cancer and received a treatment with exemestane-everolimus during the trial.
Recruitment	Patients enrolled in this trial were diagnosed with estrogen-receptor positive (ER+), HER2-negative breast cancer, refractory to non-steroidal aromatase inhibitors and eligible for exemestane-everolimus treatment according to investigator's assessment.<br>Patients were required to have at least one 18F-FDG-PET/CT evaluable lesion at baseline. This meant having a marked accumulation of 18F-FDG, at least 1.5-fold greater than standard uptake value (SUV) mean + 2 SDs in a 3-cm spherical region of interest (ROI) in a normal right lobe of liver; if the liver was abnormal, the target lesion should have had uptake > 2.0 x SUV

mean + 2 SDs of blood pool in 1-cm-diameter ROI in the descending thoracic aorta.
Sixty-four patients were screened to include 47 evaluable patients according to the 18F-FDG-PET/CT eligibility criteria.

Ethics oversight

The trial was approved by the institutional review boards and ethical committees of each participating center, and it was registered in the European Union Drug Regulating Authorities Clinical Trials Database (EudraCT number 2012-004860-22). Written informed consent was obtained from all patients before enrolment.

Note that full information on the approval of the study protocol must also be provided in the manuscript.

Clinical data

Policy information about clinical studies

All manuscripts should comply with the ICMJE guidelines for publication of clinical research and a completed CONSORT checklist must be included with all submissions.

Clinical trial registration

NCT02028364

Study protocol

The full trial protocol will be sent upon request

Data collection

Patients were recruited in 5 centers across Belgium between February 2014 and June 2018. Data collection was realized via a paper case report form during recruitment period and thereafter until the end of the study (see definition below). The analysis of the primary endpoint (progression free survival) was realized after 42 PFS events have occurred. The end of study was defined as a survival follow-up of 3 years of the last subject included or when all overall survival event were reported whichever occurred first. The database has been fully cleaned and frozen for this analysis.

Outcomes

The primary objective of the trial was to evaluate whether early metabolic response is associated with progression free survival (PFS). All 18F-FDG-PET/CT images were evaluated by two nuclear medicine specialists blinded to the clinical data. A patient was considered to be a “responder” when a uniform SUVmax reduction of more than 25% was seen in all lesions (so called consistent patient-based response). All cases not fulfilling this criterion were classified as “non-responders”. We performed a "post-hoc "analysis defining as responders those patients who showed a uniform reduction of 15% of all lesions. PFS was defined as the time elapsed between the early 18F-FDG-PET/CT (D14) and RECIST 1.1 progression, using using classical radiological assessments (CT or MRI) every 12 weeks.

The aim of the translational research in our study was to explore whether ctDNA detection at baseline or D14, or ctDNA changes between baseline and D14 were associated with PFS. Additionally, we aimed to explore whether combining 18F-FDG-PET/CT and ctDNA analysis could improve our ability to identify early on patients who will not benefit from EXE+EVE. Finally, the ctDNA analyses aimed to characterize genomic alterations in ctDNA that emerge at D14 and at progression.
